# Supplementary material for: Synthesis of Highly Porous Graphene Oxide–PEI Foams for Enhanced Sound Absorption in High-Frequency Regime
Source: Polymers (Basel). 2024 Oct 24;16(21):2983. doi: 10.3390/polym16212983 (PMC11548673; doi:10.3390/polym16212983)
Supplement: Supplementary file 1 [file polymers-16-02983-s001.zip › polymers-3264317-supplementary.pdf]

Supplementary Materials

Synthesis of Highly Porous Graphene Oxide–PEI Foams for Enhanced Sound Absorption in High-Frequency Regime

Seung-Chan Jung [1,†], Wonjun Jang [1,†], Byeongji Beom [1], Jong-Keon Won [1], Jihoon Jeong [1], Yu-Jeong Choi [1], Man-Ki Moon [1], Eou-Sik Cho [2], Keun-A Chang [3] and Jae-Hee Han [1,*]

[1] Department of Materials Science and Engineering, Gachon University, Seongnam 13120, Republic of Korea

[2] Department of Electronic Engineering, Gachon University, Seongnam 13120, Republic of Korea

[3] Department of Pharmacology, College of Medicine, Gachon University, Incheon 21999, Republic of Korea

* Correspondence: jhhan388@gachon.ac.kr (J.-H.H.); Tel.: (+82-31-750-8689)

† These authors contributed equally to this work.

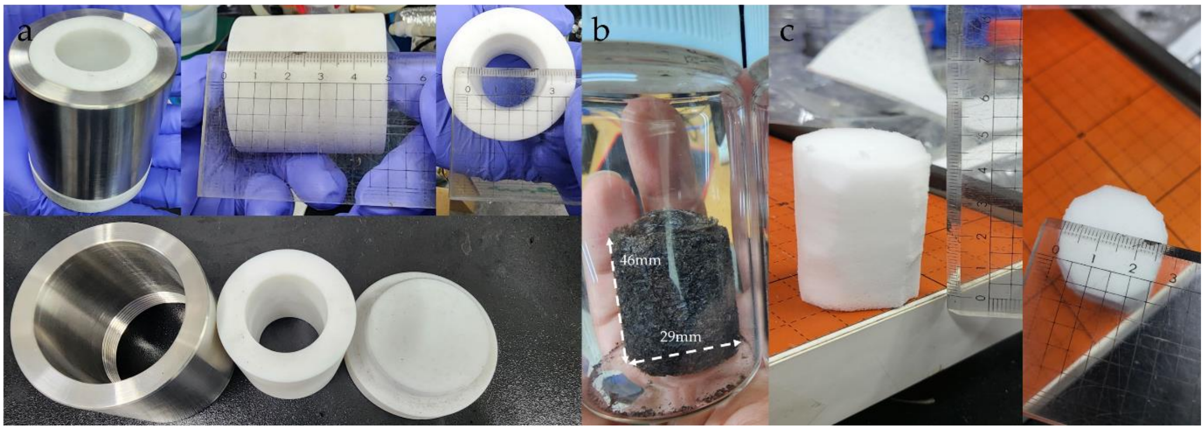

Figure S1. Images of the mold used for GPF fabrication (a) and the GPF (1:3) (b) and the melamine foam (c) samples utilized for sound absorption measurements.

Table S1. Optimized Johnson-Champoux-Allard (JCA) model parameters for the GO-PEI foam, including flow resistivity (σ), tortuosity (α_∞), porosity (Φ), viscous characteristic length (Λ), and thermal characteristic length (Λ'), derived from fitting the experimental sound absorption data (refer to Figure 7 in the main text).

Flow Resistivity (σ)	55.7E+01
Tortuosity (α_∞)	2.71E+00
Porosity (Φ)	6.09E-02
Viscous Char. Length (Λ)	3.44E-05
Thermal Char. Length (Λ')	2.14E-05

Comparison with Delany-Bazley and Miki models:

For comparison, we also applied the Delany-Bazley and Miki models (Figure 1 in the main text), which are traditionally used for simpler porous materials. These models, which mainly rely on flow resistivity σ, provided significantly poorer fits to the experimental data, as shown by the green dashed line (Delany-Bazley) and the red dotted line (Miki) in the graph. As indicated in the statistical comparison (Figure S2), the coefficient of determination R^2 values for these models were approximately -65, indicating that they are inadequate for capturing the nuanced acoustic behavior of our GO-PEI foam [39–45].

```
Statistical Comparison of Model Fits:
-------------------------------------
Model                | RMSE     | MAE      | R^2
-------------------------------------
JCA Model            | 0.0732   | 0.0586   | 0.5006
Delany-Bazley Model  | 0.8430   | 0.8366   | -65.2115
Miki Model           | 0.8430   | 0.8366   | -65.2131
-------------------------------------
```

Figure S2. Statistical comparison of model fits for the JCA, Delany-Bazley, and Miki models, summarizing the Root Mean Square Error (RMSE), Mean Absolute Error (MAE), and Coefficient of Determination R^2 values for each model.
